# Geographical variation in treated psychotic and other mental disorders in Finland by region and urbanicity

**DOI:** 10.1007/s00127-023-02516-x

**Published:** 2023-06-13

**Authors:** Kimmo Suokas, Olli Kurkela, Jaakko Nevalainen, Jaana Suvisaari, Christian Hakulinen, Olli Kampman, Sami Pirkola

**Affiliations:** 1 Faculty of Social Sciences, Tampere University, Tampere, Finland Arvo Ylpön katu 34 (Arvo 1), 33014; 2 National Institute for Health and Welfare, Helsinki, Finland; 3 Laurea University of Applied Sciences, Vantaa, Finland; 4 Department of Psychology and Logopedics, Faculty of Medicine, University of Helsinki, Helsinki, Finland; 5 Department of Health and Social Care Systems, National Institute for Health and Welfare, Helsinki, Finland; 6 Faculty of Medicine and Health Technology, Tampere University, Tampere, Finland; 7 Department of Psychiatry, The Pirkanmaa Wellbeing Services County, Tampere, Finland; 8 Department of Clinical Sciences, Psychiatry, Umeå University, Umeå, Sweden; 9 Faculty of Medicine, Department of Clinical Medicine (Psychiatry), University of Turku, Turku, Finland; 10 Department of Psychiatry, The Wellbeing Services County of Ostrobothnia, Seinäjoki, Finland; 11 Tampere University Hospital, Tampere, Finland

**Keywords:** Mental disorders, Schizophrenia, Prevalence, Geographical, Urbanicity, Social determinants

## Abstract

**Purpose:**

In Finland, prevalence of schizophrenia is higher in the eastern and northern regions and co-occurs with the distribution of schizophrenia polygenic risk scores. Both genetic and environmental factors have been hypothesized to contribute to this variation. We aimed to examine the prevalence of psychotic and other mental disorders by region and degree of urbanicity, and the impacts of socio-economic adjustments on these associations.

**Methods:**

Nationwide population registers from 2011 to 2017 and healthcare registers from 1975 to 2017. We used 19 administrative and three aggregate regions based on the distribution of schizophrenia polygenic risk scores, and a seven-level urban–rural classification. Prevalence ratios (PRs) were calculated by Poisson regression models and adjusted for gender, age, and calendar year (basic adjustments), and Finnish origin, residential history, urbanicity, household income, economic activity, and physical comorbidity (additional adjustments) on an individual level. Average marginal effects were used to visualize interaction effects between region and urbanicity.

**Results:**

A total of 5,898,180 individuals were observed. All mental disorders were slightly more prevalent (PR 1.03 [95% CI, 1.02–1.03]), and psychotic disorders (1.11 [1.10–1.12]) and schizophrenia (1.19 [1.17–1.21]) considerably more prevalent in eastern and northern than in western coastal regions. After the additional adjustments, however, the PRs were 0.95 (0.95–0.96), 1.00 (0.99–1.01), and 1.03 (1.02–1.04), respectively. Urban residence was associated with increased prevalence of psychotic disorders across all regions (adjusted PR 1.21 [1.20–1.22]).

**Conclusion:**

After adjusting for socioeconomic and sociodemographic factors, the within-country distribution of mental disorders no longer followed the traditional east–west gradient. Urban–rural differences, on the other hand, persisted after the adjustments.

**Supplementary Information:**

The online version contains supplementary material available at 10.1007/s00127-023-02516-x.

## Introduction

The prevalence of psychotic and other mental disorders varies globally and locally [1–4], with urban–rural differences being a particularly important factor in Northern Europe [5–9]. The underlying mechanisms for these variations are not well understood and are thought to be influenced by a combination of neighbourhood and individual-level social-environmental factors, including pollution, lack of green space, social stress or selective migration, among other things [5]. Some combined analyses have shown gene-environment synergism in the risk profiles [10–14].

In Finland, there is a well-documented pattern of higher prevalence of schizophrenia and other psychotic disorders in the east and of mood and anxiety disorders in the south [15–20]. In schizophrenia, regional differences have been more significant than urban–rural variations, and this geographical east–west pattern in schizophrenia prevalence coincides with schizophrenia polygenic risk scores, leading to the hypothesis that population genetics may play a role (Supplementary Fig. S1a) [16, 17, 21, 22]. However, social determinants of mental health, such as the proportion of low-income earners (Supplementary Fig. S1b), level of education, unemployment, migration, or household structure also vary across the country with less favourable compositions often seen in the eastern parts of the country. Urban areas, on the other hand, are more common in southern and western regions (Supplementary Fig. S1c). It is not known to what extent regional and urban–rural variations interact, and to what extent the geographical variations are confounded by socioeconomic factors.

**Fig. 1:**
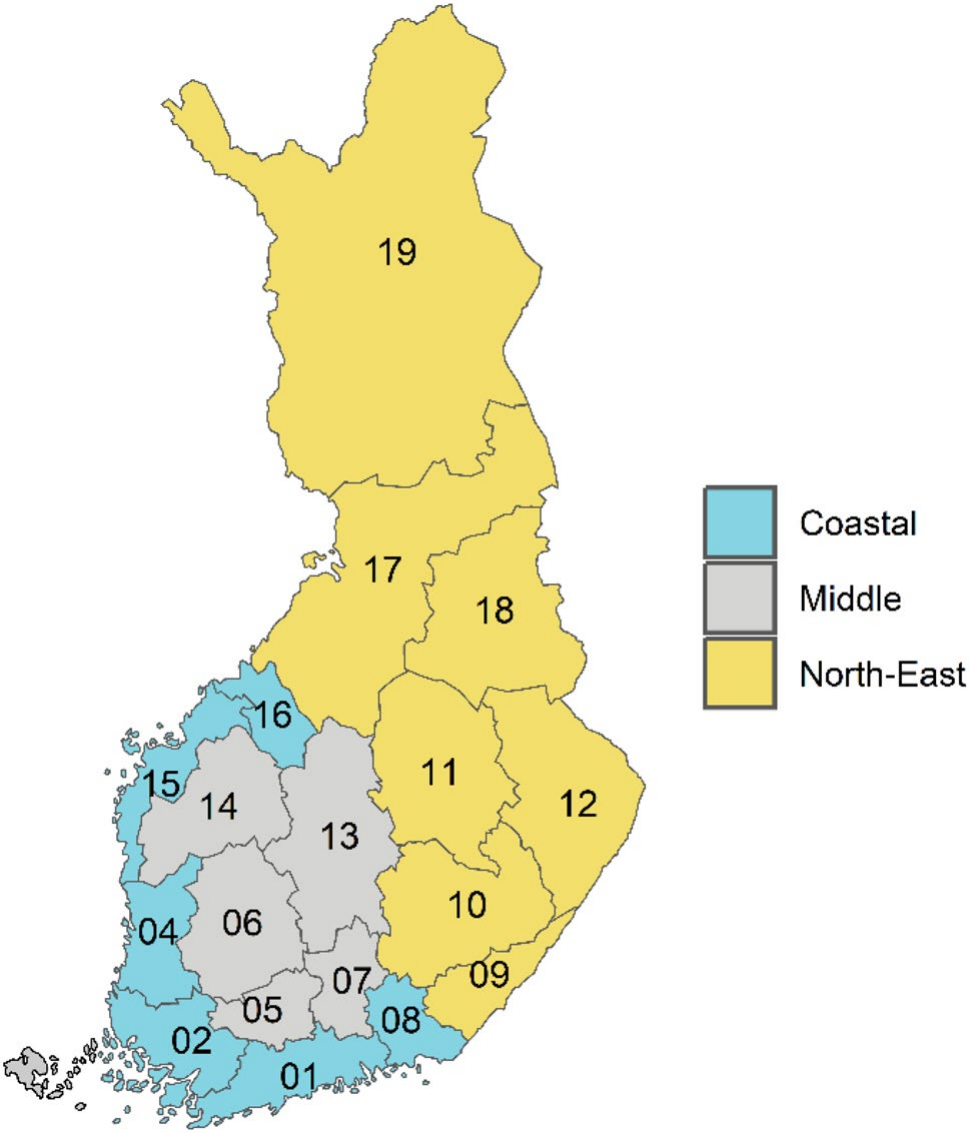
Administrative regions in Finland and aggregate regions based on the polygenic risk-score distribution in this study. Regions: 01 Uusimaa, 02 Varsinais-Suomi, 04 Satakunta, 05 Kanta-Häme, 06 Pirkanmaa, 07 Päijät-Häme, 08 Kymenlaakso, 09 South Karelia, 10 Etelä-Savo, 11 Pohjois-Savo, 12 North Karelia, 13 Central Finland, 14 South Ostrobothnia, 15 Ostrobothnia, 16 Central Ostrobothnia, 17 North Ostrobothnia, 18 Kainuu, 19 Lapland

We aimed to evaluate regional and urban–rural variation in psychotic and all mental disorders, their interaction, and the impact of socioeconomic adjustments on these geographical differences. To facilitate comparisons of geographical differences in prevalence of schizophrenia with different adjustments and schizophrenia polygenic risk scores that have previously been reported, we grouped the administrative regions of Finland into three aggregate regions and aimed to present detailed maps of the geographical prevalence distributions (Fig. 1). We hypothesized that much of the variability in prevalence of mental disorders would be explained by demographic and socioeconomic factors.

## Methods

We conducted a population-based register study including all individuals living in Finland from 2011 to 2017. Using individual-level population and health care registers, we calculated the prevalence of people with a history of mental health-related contact with primary care or psychiatric secondary inpatient or outpatient care on the last day of each of the study years. In addition, all individuals living in Finland between 1996 and 2017 were followed up in the registers to identify the incidence of the first psychiatric inpatient admissions. These time limits were based on the coverage of the national health care registers.

The Research Ethics Committee of the Finnish Institute for Health and Welfare approved the study protocol (decision #10/2016§751). Data were linked with permission from Statistics Finland (TK-53–1696-16) and the Finnish Institute of Health and Welfare. Informed consent is not required for register-based studies in Finland.

### Assessment of mental disorders

Information on mental healthcare was obtained from the Finnish Care Register for Health Care. Psychiatric inpatient care can be reliably recognized since 1975, secondary outpatient care has been included since 2006 and primary care has been included since 2011 (for details, see Supplementary Methods).

The International Statistical Classification of Diseases and Related Health Problems, Tenth Revision (ICD-10) has been used in Finland since 1996. We described specific disorders with the ten-level ICD-10 sub-chapter categories and in the following categories: all psychotic disorders (ICD-10: F20-29, F30.1, F30.2, F30.8, F30.9, F31.1, F31.2, F31.5, F31.6, F32.3, F33.3, F1x.5, F1x.7), mania and bipolar disorders with psychotic symptoms (F30.1, F30.2, F30.8, F30.9, F31.1, F31.2, F31.5, F31.6), psychotic depression (F32.3, F33.3), and substance-induced psychotic disorders (F1x.5, F1x.7). The diagnoses of schizophrenia and other primary psychotic disorders were classified in a particular order, with schizophrenia being the first (F20), followed by schizoaffective disorder (F25), delusional disorders (F22 and F24), brief psychotic disorders (F23), schizotypal disorder (F21), other nonorganic psychotic disorders (F28), and unspecified nonorganic psychosis (F29). If a person had more than one diagnosis from the schizophrenia spectrum, they were classified under the first group of disorders in the order presented above.

In primary care, the ICPC-2 International Classification of Primary Care, instead of ICD-10, is used in some facilities, and ICPC-2 mental health-related diagnoses were converted to corresponding ICD-10 sub-chapter categories when possible (for details, see Supplementary Methods).

Discharge diagnoses and diagnoses from outpatient visits were also collected. A description of the method used for handling partly overlapping register data entries is publicly available [23].

### Regions and urban–rural classification

Finland consists of 19 administrative regions, each with a central town, possible other towns and surrounding areas with varying degrees of urbanicity. Based on the distribution of the schizophrenia polygenic risk score [21, 22], we grouped the administrative regions into three aggregate regions: coastal, inland, and eastern and northern (Fig. 1). The region of residence on the last day of each study year was used for the main analysis. We used the seven-level urban–rural classification for the year 2010 issued by the Finnish Environment Institute based on a nationwide grid of 250 × 250 m cells, to measure urbanicity for each individual's place of residence [24]. In order to show geographical variation by region and urbanicity, we created maps with region-urbanicity subregions (Supplementary Fig. S1c).

### Cofactors

We collected the following categorical individual-level demographic and socioeconomic data on the last day of each study year from the population registers: age (five-year intervals), gender (man or woman), origin (Finnish background or not, determined based on the country of birth data of the person's parents [25]), currently inhabiting the region of birth (yes or no), economic activity (employed; unemployed; students; pensioners and others outside the labour force), and equivalized household net income deciles. Net income was obtained after subtracting taxes and was adjusted for the size of the household dwelling unit using the Organisation for Economic Cooperation and Development–modified equivalence scale.

Physical comorbidity was assessed using the Charlson comorbidity index (CCI), a widely used comorbidity index with a weighted score of 17 comorbid conditions [26]. For each study year and for every individual in the study, the CCI score was calculated using available ICD-10 diagnoses of any actual treatment contact in healthcare registers from the beginning of the previous calendar year. Age was not included in the CCI scores but was adjusted in the main model. CCI scores were categorized by previously used cut-points: none, 1–3, and ≥ 4 [27].

### Statistical analysis

The prevalence of a history of mental disorders was calculated for the last day of each calendar year of the study by summing the number of people with a history of mental health treatments in each region divided by the number of inhabitants in the region. Data were aggregated by strata defined by all possible combinations of cofactors. Prevalence ratios were examined using a Poisson regression model with a robust sandwich variance estimator. The strata in the aggregated data were taken as the unit of analysis and the log of population size of the strata was used as an offset term.

Regional prevalence ratios were adjusted for gender, age, and calendar year (basic adjustment). Additional adjustments for origin, residential history, urbanicity, household income, economic activity, and CCI were also made. Bayesian information criteria were used for the model selection.

For a fine-scale view of the variability of prevalence by region and urbanicity, the average marginal effects for each region-urbanicity subregion were predicted using a Poisson regression model that included a region-urbanicity interaction term. The predicted prevalence in each region-urbanicity subregion was calculated while holding the other predictors constant as observed [28].

The sensitivity to definitions of the outcome and explanatory variables was investigated by alternative definitions and comparison of results across the following additional analyses: The prevalence of all treated mental disorders and inpatient treatments only were compared; the incidence and prevalence of regional inpatient treatments were compared; and the current living region and the region of birth were compared. For data management and analyses, we used R, version 3.6.3 (R Project for Statistical Computing), and Stata, version 17.1 (StataCorp LLC).

## Results

During the years 2011 to 2017, a total of 5,898,180 individuals contributed to the study population. Altogether, 1,197,690 individuals of the total of 5,512,745 at the end of 2017 had a history of some medical contact in primary or secondary care mental health services. This resulted in a crude prevalence rate of 21.73% (24.07% in women and 19.32% in men). Prevalences stratified by the covariates are reported in the Supplementary Table.

### Regional variation in prevalence of mental disorders

The crude prevalence of all psychotic disorders, schizophrenia, and most of the other psychotic disorders was higher in the eastern and northern than in the coastal regions (Table 1). However, unspecified psychosis, bipolar disorder and substance-induced psychotic disorders, as well as mood disorders and neurotic disorders, were more common in the coastal region, resulting in only a minimal difference in the prevalence of all mental disorders (Table 1).

**Table 1:** Prevalence of mental disorders by place of residence in 2017: number of cases, prevalence rates, and crude prevalence ratios (PR)^a^

	Number of diagnosed individuals (prevalence %)				PR (95% CI)
	Whole country 5 512 745 (100%)^b^	Coastal 2 808 181 (50.9%)^b^	Inland 1 352 887 (24.5%)^b^	Eastern and northern 1 351 677 (24.5%)^b^	Eastern and northern vs. coastal
Any mental disorder (F00-99)	1 197 690 (21.73)	599 739 (21.36)	302 794 (22.38)	295 157 (21.84)	1.02 (1.01–1.02)
All psychotic disorders^c^	112 318 (2.04)	55 722 (1.98)	26 456 (1.96)	30 140 (2.23)	1.10 (1.09–1.12)
Schizophrenia spectrum (F20-29)^d^	93 182 (1.69)	44 537 (1.59)	22 448 (1.66)	26 197 (1.94)	1.21 (1.20–1.22)
Schizophrenia (F20)	34 269 (0.62)	16 567 (0.59)	7 821 (0.58)	9 881 (0.73)	1.19 (1.17–1.21)
Schizoaffective disorders (F25)	6 720 (0.12)	3 141 (0.11)	1 645 (0.12)	1 934 (0.14)	1.26 (1.23–1.29)
Delusional disorders (F22, F24)	11 092 (0.20)	5 156 (0.18)	2 972 (0.22)	2 964 (0.22)	1.15 (1.13–1.17)
Brief psychotic disorders (F23)	8 830 (0.16)	4 459 (0.16)	2 303 (0.17)	2 068 (0.15)	1.00 (0.98–1.02)
Schizotypal disorder (F21)	2 458 (0.04)	1 070 (0.04)	583 (0.04)	805 (0.06)	1.59 (1.53–1.65)
Other (F28)	1 009 (0.02)	441 (0.02)	269 (0.02)	299 (0.02)	1.33 (1.25–1.41)
Unspecified (F29)	17 238 (0.31)	8 953 (0.32)	4 062 (0.30)	4 223 (0.31)	0.97 (0.95–0.99)
Bipolar disorder^e^	44 890 (0.81)	24 438 (0.87)	10 512 (0.78)	9 940 (0.74)	0.82 (0.81–0.83)
Psychotic depression^f^	22 167 (0.40)	10 929 (0.39)	4 869 (0.36)	6 369 (0.47)	1.19 (1.17–1.21)
Substance-induced psychotic disorders^g^	9 672 (0.18)	5 295 (0.19)	2 101 (0.16)	2 276 (0.17)	0.88 (0.86–0.90)
Substance use disorders (F10-19)	161 307 (2.93)	81 372 (2.90)	38 485 (2.84)	41 450 (3.07)	1.04 (1.03–1.05)
Mood disorders (F30-39)	416 542 (7.56)	214 956 (7.65)	105 926 (7.83)	95 660 (7.08)	0.92 (0.91–0.92)
Neurotic disorders (F40-48)	460 247 (8.35)	239 839 (8.54)	115 580 (8.54)	104 828 (7.76)	0.87 (0.87–0.88)

^a^Prevalence of a history of treated disorders on 31 Dec 2017. The aggregate regions are described in Fig. 1

^b^Total population in the region (percentage of whole country population)

^c^All psychotic disorders included the following disorders: schizophrenia spectrum disorders (F20-29), mania and bipolar disorder with psychotic symptoms (F30.1, F30.2, F30.8, F30.9, F31.1, F31.2, F31.5, F31.6), psychotic depression (F32.3, F33.3), and substance-induced psychotic disorders (F1x.5 through F1x.7)

^d^Schizophrenia spectrum diagnoses were categorized in the order presented in the table

^e^Bipolar disorder included mania and bipolar disorder with psychotic symptoms (F30.1, F30.2, F30.8, F30.9, F31.1, F31.2, F31.5, F31.6)

^f^Psychotic depression included diagnoses F32.3 and F33.3

^g^Substance-induced psychotic disorders included three categories of substance-induced psychotic disorders (F1x.5 through F1x.7)

**Fig. 2:**
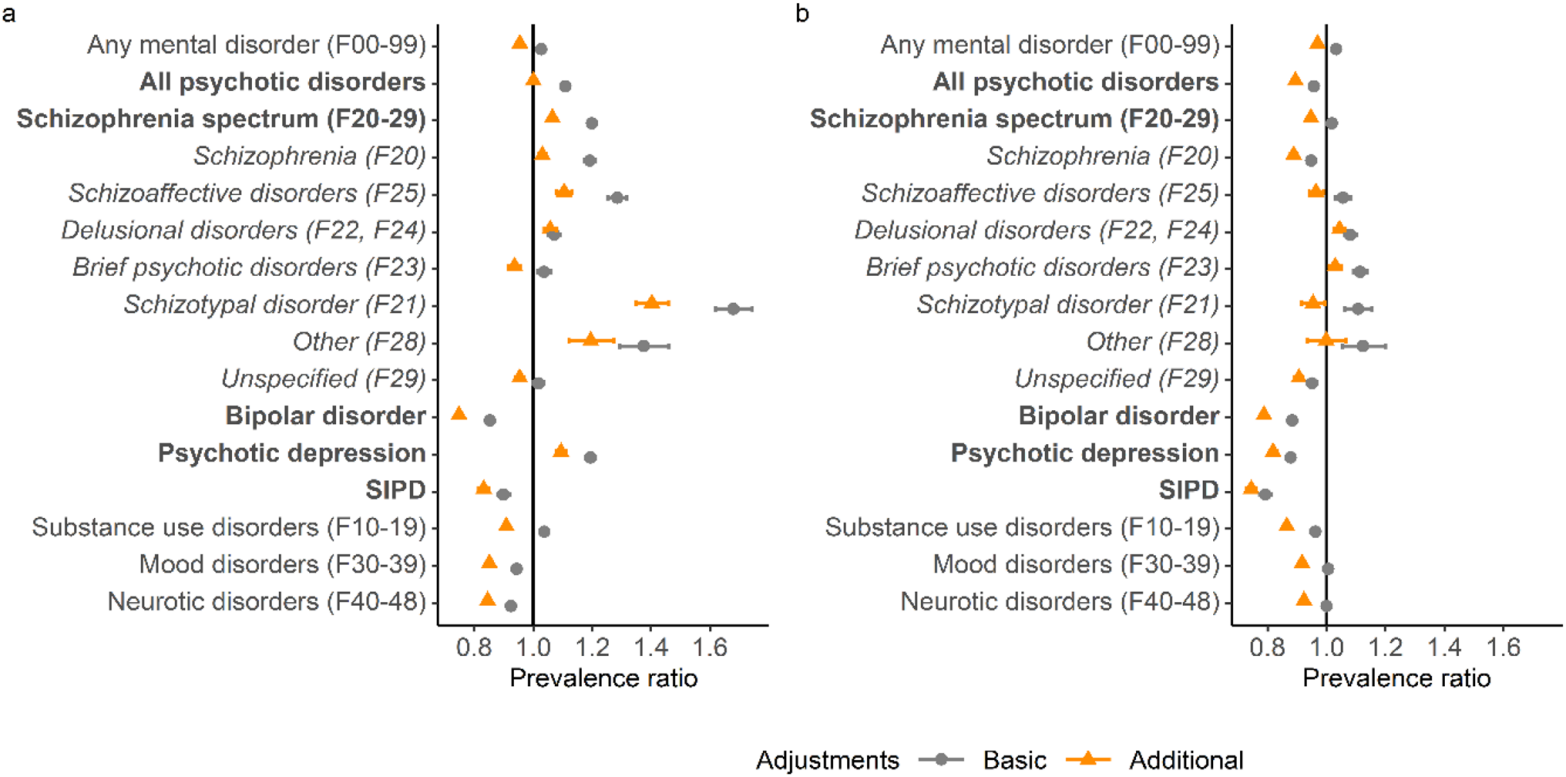
Prevalence ratios of mental disorders by place of residence. Higher prevalence ratios indicate higher risk in **a** eastern and northern and **b** inland regions compared to coastal regions

After basic adjustments, prevalence ratios (PRs) of 1.11 (95% CI 1.10–1.12) for all psychotic disorders, 1.20 (1.19–1.21) for schizophrenia spectrum, 0.85 (0.84–0.86) for bipolar disorder, and 1.03 (1.02–1.03) for all mental disorders in the eastern and northern compared to the coastal regions were observed (Fig. 2a).

Coastal, Inland, and East-north regions are described in Fig. 1. In the basic adjustment, prevalence ratios are adjusted for age, gender, and calendar time. In the additional adjustment, prevalence ratios were adjusted for age, gender, calendar time, urbanicity, origin, residence history, household income, economic activity, and Charlson comorbidity index. Error bars indicate 95% Cis. Subgroups of all included psychotic disorders are highlighted in bold. Bipolar disorder included ICD-10 codes F30.1, F30.2, F30.8, F30.9, F31.1, F31.2, F31.5, F31.6, psychotic depression codes F32.3 and F33.3, and substance-induced psychotic disorders (SIPD) codes F1x.5 to F1x.7. Schizophrenia spectrum diagnoses (in italic) were categorized in the order presented in the figure.

When additional adjustments for socioeconomic factors and comorbidities were included in the models, the eastern and northern prominence in psychotic disorders disappeared, with a PR of 1.00 (0.99–1.01). PRs of 1.06 (1.06–1.07) for schizophrenia spectrum, 1.03 (1.02–1.04) for schizophrenia, and 0.75 (0.74–0.76) for bipolar disorder were observed (Fig. 2a). The PR for all mental disorders was 0.95 (0.95–0.96) (Fig. 2a). Adding income to the models caused a major change in the PR estimates, and the effect of each of the additional covariates is shown in the online Supplementary Fig. S2. There were some variations between neighbouring regions within the aggregate regions and between diagnoses (Supplementary Fig. S3).

### Urban–rural variation in prevalence of mental disorder

**Fig. 3:**
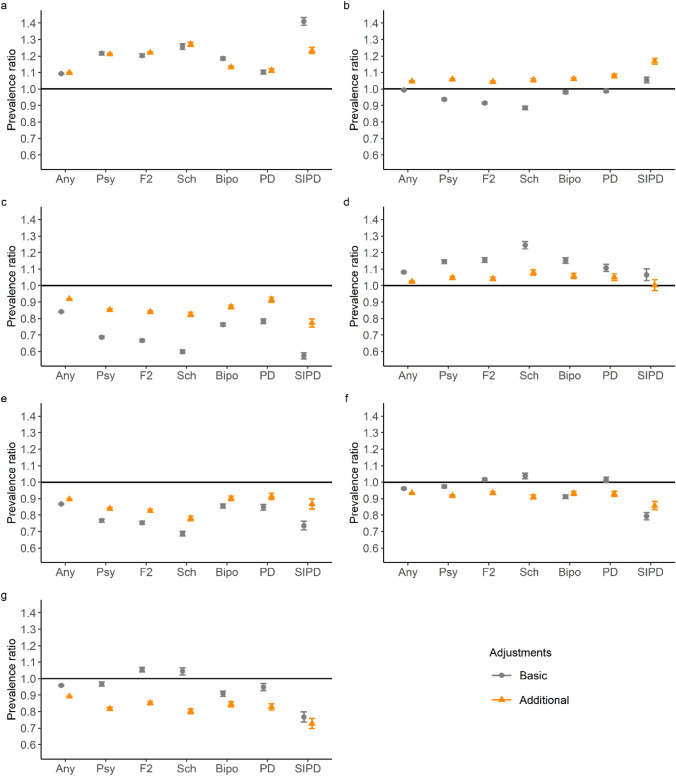
Prevalence ratios of selected mental disorders by urbanicity of the place of residence, compared to the national mean. **a** Inner urban area (32.5%) **b** Outer urban area (26.4%) **c** Peri-urban area (11.0%) **d** Local centres in rural areas (5.8%) **e** € Rural areas close to urban (7.1%) **f** Rural heartland areas (10.8%) **g** Sparsely populated rural areas (5.1%). The proportion of population living in each level of urbanicity is given in parentheses. Any refers to any mental disorder, Psy to all psychotic disorders, F2 to schizophrenia spectrum, Sch to schizophrenia, Bipo to bipolar disorder, PD to psychotic depression, and SIPD refers to substance-induced psychotic disorders. In the basic adjustment, prevalence ratios are adjusted for age, gender, and calendar time. In the additional adjustment, prevalence ratios were adjusted for age, gender, calendar time, region, origin, residence history, household income, economic activity, and Charlson comorbidity index. Error bars indicate 95% CIs

Residence in inner urban areas or in the local centres in rural areas was clearly associated with increased prevalence of all mental disorders and major psychotic disorders in both levels of adjustment (Fig. 3). The additional adjustments changed the prevalence ratios in some levels of urbanicity, although the link between urbanicity and psychotic disorders remained clear. In inner urban areas, PRs of 1.10 (1.10–1.10) for all mental disorders and 1.21 (95% CI, 1.20–1.22) for psychoses, compared to the whole national mean with additional adjustments, were observed.

### Prevalence of mental disorders by region and urbanicity

**Fig. 4:**
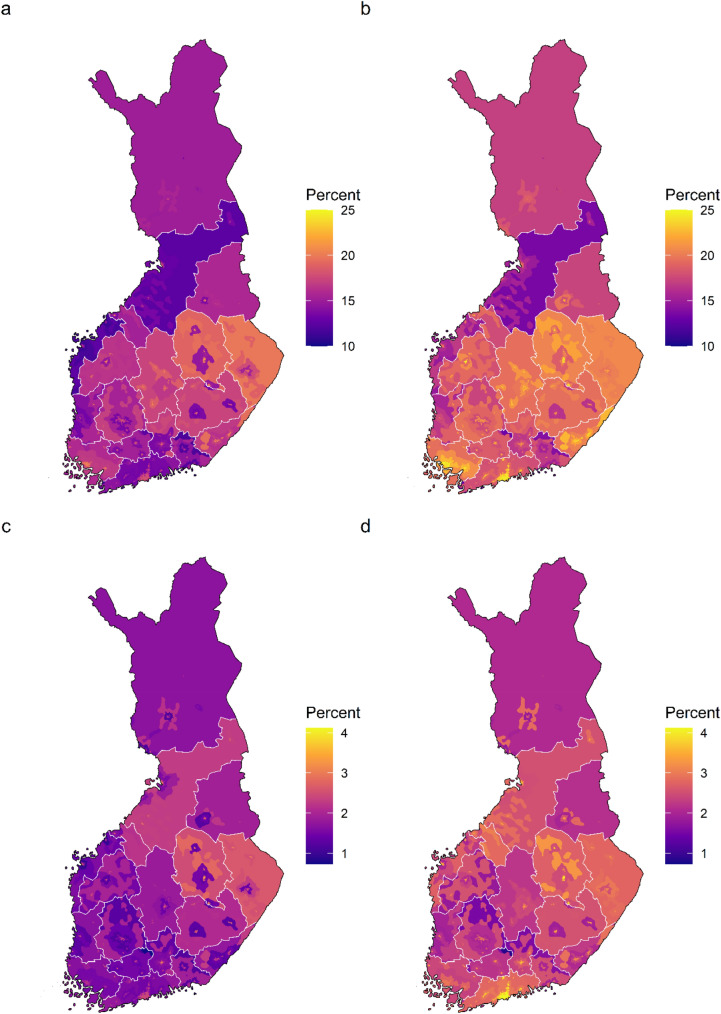
Average marginal effects of region of residence and urbanicity on the prevalence of any mental disorder and all psychotic disorders. **a** any mental disorder, basic adjustments, **b** any mental disorder, additional adjustments, **c** all psychotic disorders, basic adjustments, and **d** all psychotic disorders, additional adjustments. Predicted prevalence in each region-urbanicity subregion was calculated while holding the other predictors constant as observed. In the basic adjustment, prevalence ratios were adjusted for age, gender, and calendar time. In the additional adjustment, prevalence ratios were adjusted for age, gender, calendar time, origin, residence history, household income, economic activity, and Charlson comorbidity index

**Fig. 5:**
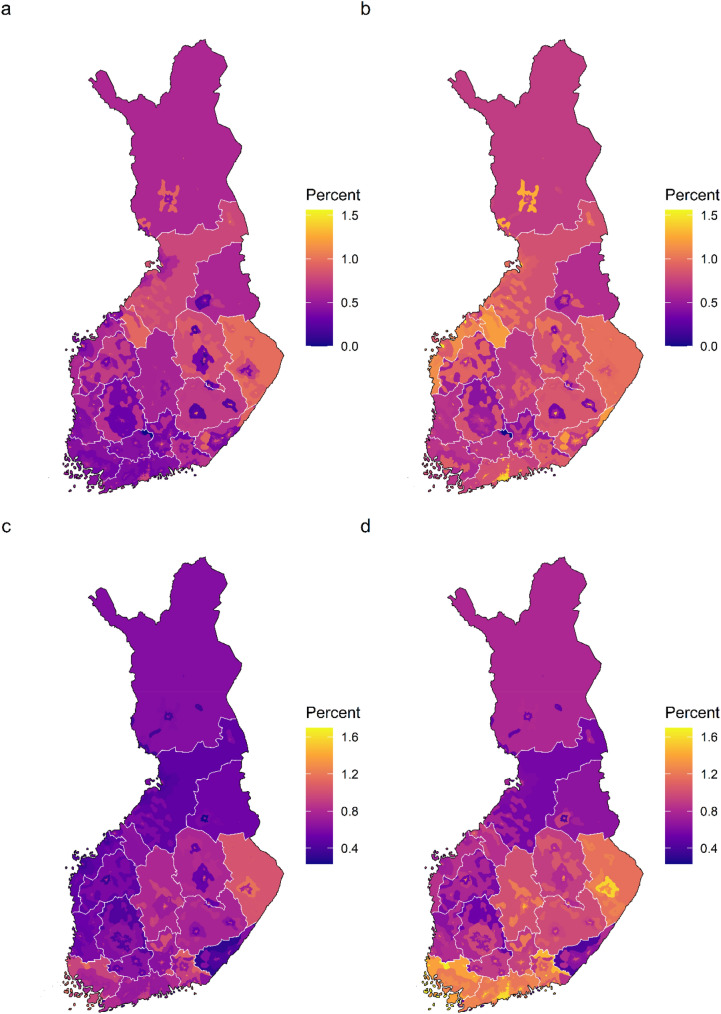
Average marginal effects of region of residence and urbanicity on the prevalence of schizophrenia and bipolar disorder. **a** schizophrenia, basic adjustments, **b** schizophrenia, additional adjustments, **c** bipolar disorder, basic adjustments, and **d** bipolar disorder, additional adjustments. Predicted prevalence in each region-urbanicity subregion was calculated while holding the other predictors constant as observed. In the basic adjustment, prevalence ratios were adjusted for age, gender, and calendar time. In the additional adjustment, prevalence ratios were adjusted for age, gender, calendar time, origin, residence history, household income, economic activity, and Charlson comorbidity index

The analysis of prevalence of mental disorders by region of residence and urbanicity with basic adjustments showed an eastern and northern prominence in the prevalence of all mental disorders and psychotic disorders in all levels of urbanicity. After the additional adjustments, prominence of the inner urban area in the coastal regions became evident across any mental disorders, all psychotic disorders, and schizophrenia. Furthermore, after the additional adjustments, bipolar disorder come up in the coastal regions in all levels of urbanicity (Supplementary Fig. S4). The average marginal effects of prevalence for each region-urbanicity subregion are visualized in the maps (Figs. 4 and 5).

### Additional analyses

The following additional analyses were conducted: First, if inpatient care was analyzed alone, clear eastern and northern prominence would have been observed (Supplementary Fig. S5). Second, irrespective of whether region of birth or region of residence was utilized as the explanatory variable, the prevalence ratios with basic adjustments revealed the prominence of eastern and northern regions in any mental disorders, all psychotic disorders, and schizophrenia (Supplementary Fig. S6). Third, using data on incidence of the first inpatient episodes instead of prevalence would cause changes in the proportions of different diagnostic categories. In inpatient treated cases of all mental disorders and all psychotic disorders, the eastern and northern prominence persisted. In the case of the schizophrenia spectrum, however, the observed difference in geographical prominence disappeared. (Supplementary Fig. S7). Fourth, the eastern and northern prominence in any mental disorder and all psychotic disorders disappeared after the additional adjustments in both men and women (Supplementary Fig. S8).

## Discussion

In this nationwide register-based study of over 5 million Finnish persons, we found that the prevalence of all mental disorders and psychotic disorders treated in both primary or secondary care was higher in the eastern and northern regions compared to coastal regions. After adjusting for socioeconomic factors, however, this geographical difference was no longer evident. By contrast, the urban–rural differences, as measured using a detailed seven-level classification of current residency, persisted after the adjustments and were consistent with previous findings from other Nordic countries. Urban effect was evident across the country and diagnostic categories, although regional differences in some diagnostic subgroups, such as schizophrenia and bipolar disorders, were observed. Taken together, our results demonstrate the significant impact of social determinants on the mental health of the population and have important national implications.

To the best of our knowledge, this is the first comprehensive study demonstrating the associations between the within-country distribution of socioeconomic and demographic factors and the prevalence of mental disorders treated with either primary or secondary care. Epidemiological studies in Finland have investigated regional and urban–rural variations in mental disorders since the 1930s but have usually included only inpatient register data [15–20, 29]. Including outpatient and primary care data can be seen as a main strength of the current study, as including this data substantially changed the prevalence ratios in eastern and norther parts of the country, and there are some variations in the overall inpatient care across the regions [30]. The east–west differences have been consistently observed, but one recent study found significant regional variation in mental disorder disability pensions that did not follow the traditional east–west health differences [31]. Thus, within-country geographical differences in mental health are sensitive to a variety of social determinants and draw a more complex picture than reported in previous studies.

Previous findings on the association between urbanicity and mental disorders in Finland have been mixed, with the earliest studies showing an association between living in cities and schizophrenia [29], but more recent studies suggestive of urban effects but yielding inconsistent results [15–17]. Current results align with previous studies in Northern Europe, demonstrating an association between variety of psychotic disorders and urbanicity [5, 7, 32]. Finland no longer appears to be an exception in this respect. Structural changes in demography, employment and services have affected particularly eastern rural parts of the country in recent decades and probably affect the temporal differences in the link between urbanicity and psychotic disorders in Finland [17, 33].

Household income was a particularly strong cofactor in the models. This is not surprising, as income inequality and individual level low income and mental disorders have been strongly linked with complex bi-directional pathways [34–38]. In the current study, we did not explore the causal pathways behind the mental disorders and income distribution. Nevertheless, income was a relevant cofactor, as it is unlikely that the within-country distribution of income was determined by the regional prevalence of mental disorders.

Contrary to regional differences, urban–rural variation did not disappear after socioeconomic adjustments. Urban environments in a sparsely populated country such as Finland may vary greatly within the country in terms of potential urban risk attributes such as nature spaces, migration, social stress, or demographical and socioeconomic composition. Our analysis of the urbanicity-region interaction with socioeconomic adjustments showed that urbanicity is a relevant factor for mental health in all regions of the country, regardless of the size of the regional urban centre, from Kajaani with a population of 36,000 to Helsinki with a population of 665,000. We evaluated regional differences and urbanicity based on current residency, while controlling for living in the birth region. This approach enabled accounting for within-country migration. However, we did not have data on individual histories of urban residency or changes in geographical distribution of urbanicity. Selective migration can affect regional composition and socioeconomic contexts, and also affects the associations between urbanicity and psychotic disorders [5, 10, 39, 40]. In Finland, however, it has been suggested that individuals with mental disorders are not particularly likely to move to the most urban centers [41], and accessibility of Finnish primary health care is mostly at good level, although in a recent study, travel time in rural areas negatively associated with primary care mental health service use [42, 43].

The relatively high prevalence of bipolar disorder with psychotic features in southern urban areas and the comparatively high prevalence of schizophrenia spectrum diagnoses in eastern and northern areas emphasize the importance of considering different register-based diagnoses side by side. Although the Finnish registers show good consistence [44], a tendency towards a narrow definition of schizophrenia in clinical practice in Finland has been recognized [45]. Whether there are differences in diagnostic practices in primary or secondary care mental health services across the country has not been evaluated recently. In Finland, there is a relatively high number of specialists in psychiatry and general practitioners are trained in psychiatry as well [46]. With the observed differences in certain diagnostic categories in mind, future assessment of the real-world diagnostic consistency and reliability might be useful in terms of both scientific and clinical accuracy.

The study of population genetics in Finland has attracted a great deal of interest, and there is a well-documented north–south and east–west genetic differentiation within the population [22, 47, 48]. Although the use of polygenic risk scores for explaining geographic differences in phenotypes is not currently recommended due to methodological limitations, the striking similarity between schizophrenia prevalence and polygenic scores has been suggested as an example of the potential of polygenic risk scores to explain geographic health differences [21]. Our results showed that after adjusting for socioeconomic factors, the prevalence of all psychotic disorders did not display statistically significant east–west differences, and did not align with the geographical gradient of schizophrenia polygenic scores. A diagnosis of schizophrenia was slightly more prevalent in eastern parts of the country, but did not follow a gradient that was comparable to that of schizophrenia polygenic scores. Mental disorders are highly polygenic and pleiotrophic, and most of their genetic common variant architecture has not been identified [49]. Schizophrenia polygenic risk scores are associated with a variety of traits, adding complexity to the concept [50–54]. In the present study, however, genetics were not evaluated. Thus, accounting for neighbourhood contextual factors and socioeconomic composition and individual level social determinants, together with genetic information, may be beneficial in future studies of geographical differences in mental health in Finland.

### Strengths and limitations

The main strength of our study is the use of interlinked Finnish national registers, which provide comprehensive data on both primary and secondary care treatments for mental disorders across the country. The inclusion of primary care treatment data is important, as primary care mental health treatment is common in Finland, and our previous study showed that including primary care may alter findings [55]. There is no universal definition of urbanicity, and to the best of our knowledge the current seven-level classification with 250 × 250 m pixels has not been used before in this context and is more detailed than previous classifications.

This study has certain limitations. First, primary care data is available only since 2011, and to our knowledge, there are no studies on the accuracy of primary care psychiatric diagnoses in the Finnish registers. Hence, incident cases cannot be recognized. The prevalence of treated mental health treatments was the outcome of interest, and we did not focus on the complex bi-directional causal chains of income and mental health on an individual level, but rather on the overall composition of the population. Second, the current urban–rural classification is available only since 2010, and therefore historical changes in urban effects cannot be evaluated and the individual level residence history by urbanicity cannot by traced. Third, no individual level genetic data was used and thus the comparison between our study and that of Kurki et al. is indirect [22]. Fourth, private and employer-paid mental health outpatient care are significant components of the Finnish health care system, and probably more common in urban settings, but were not covered in the registers for the study period. Finally, the present observational results do not allow a causal interpretation.

## Conclusion

Urbanicity and socioeconomic position are important determinants of geographical variations in population mental health. In this study, the previously well documented east–west gradient in psychotic disorders that coincides with the geographical distribution of schizophrenia polygenic risk scores, was no longer observed after detailed adjustments. Our current findings align with previous studies in Northern Europe, demonstrating a solid association between psychotic disorders and urbanicity also in Finland, which has previously been uncertain. At the national level, acknowledging these geographical patterns and their correlations with societal factors may enhance understanding of population health. While the utilization of primary care registers represents a noteworthy strength for Finnish register-based epidemiology, their diagnostic accuracy regarding mental disorders remains to be evaluated. Further study is needed to provide better understanding of the geographical patterns of mental health.

### Supplementary Information

Below is the link to the electronic supplementary material.

Supplementary file1 (PDF 3626 KB)

## Data Availability

The data that support the findings of this study are available from the National Institute of Health and Welfare (http://www.thl.fi) and Statistics Finland (www.stat.fi). Restrictions apply to the availability of these data, which were used under license for this study. Inquiries about secure access to data should be directed to data permit authority Findata (https://findata.fi/en/). A description of the method used for handling partly overlapping register data entries is publicly available [23].

## References

[1] Jongsma HE, Gayer-Anderson C, Lasalvia A (2018). Treated incidence of psychotic disorders in the multinational EU-GEI study. JAMA Psychiat.

[2] Simeone JC, Ward AJ, Rotella P (2015). An evaluation of variation in published estimates of schizophrenia prevalence from 1990–2013: a systematic literature review. BMC Psychiatry.

[3] Steel Z, Marnane C, Iranpour C (2014). The global prevalence of common mental disorders: a systematic review and meta-analysis 1980–2013. Int J Epidemiol.

[4] GBD 2019 Mental Disorders Collaborators (2022). Global, regional, and national burden of 12 mental disorders in 204 countries and territories, 1990–2019: a systematic analysis for the Global Burden of Disease Study 2019. Lancet Psychiatry.

[5] Krabbendam L, Van Vugt M, Conus P (2021). Understanding urbanicity: how interdisciplinary methods help to unravel the effects of the city on mental health. Psychol Med.

[6] Pedersen CB, Antonsen S, Timmermann A (2022). Urban-rural differences in schizophrenia risk: multilevel survival analyses of individual- and neighborhood-level indicators urbanicity and population density in a danish national cohort study. Schizophr Bull Open.

[7] Vassos E, Agerbo E, Mors O, Bøcker Pedersen C (2016). Urban–rural differences in incidence rates of psychiatric disorders in Denmark. Br J Psychiatry.

[8] Fett A-KJ, Lemmers-Jansen ILJ, Krabbendam L (2019). Psychosis and urbanicity: a review of the recent literature from epidemiology to neurourbanism. Curr Opin Psychiatry.

[9] Abrahamyan Empson L, Baumann PS, Söderström O (2020). Urbanicity: The need for new avenues to explore the link between urban living and psychosis. Early Interv Psychiatry.

[10] Colodro-Conde L, Couvy-Duchesne B, Whitfield JB (2018). association between population density and genetic risk for Schizophrenia. JAMA Psychiatry.

[11] Fan CC, McGrath JJ, Appadurai V (2018). Spatial fine-mapping for gene-by-environment effects identifies risk hot spots for schizophrenia. Nat Commun.

[12] Maxwell JM, Coleman JRI, Breen G, Vassos E (2021). Association between genetic risk for psychiatric disorders and the probability of living in urban settings. JAMA Psychiat.

[13] Paksarian D, Trabjerg BB, Merikangas KR (2018). The role of genetic liability in the association of urbanicity at birth and during upbringing with schizophrenia in Denmark. Psychol Med.

[14] Robinson N, Bergen SE (2021). Environmental risk factors for schizophrenia and bipolar disorder and their relationship to genetic risk: current knowledge and future directions. Front Genet.

[15] Suvisaari J, Opler M, Lindbohm M-L, Sallmén M (2014). Risk of schizophrenia and minority status: a comparison of the Swedish-speaking minority and the Finnish-speaking majority in Finland. Schizophr Res.

[16] Perälä J, Saarni SI, Ostamo A (2008). Geographic variation and sociodemographic characteristics of psychotic disorders in Finland. Schizophr Res.

[17] Haukka J, Suvisaari J, Varilo T, Lnnqvist J (2001). Regional variation in the incidence of schizophrenia in Finland: a study of birth cohorts born from 1950 to 1969. Psychol Med.

[18] Korkeila JA, Lehtinen V, Tuori T, Helenius H (1998). Regional differences in the use of psychiatric hospital beds in Finland: a national case-register study. Acta Psychiatr Scand.

[19] Hovatta I, Terwilliger JD, Lichtermann D (1997). Schizophrenia in the genetic isolate of Finland. J Med Genet.

[20] Lehtinen V, Joukamaa M, Lahtela K (1990). Prevalence of mental disorders among adults in Finland: basic results from the Mini Finland Health Survey. Acta Psychiatr Scand.

[21] Kerminen S, Martin AR, Koskela J (2019). Geographic variation and bias in the polygenic scores of complex diseases and traits in Finland. Am J Hum Genet.

[22] Kurki MI, Saarentaus E, Pietiläinen O (2019). (2019) Contribution of rare and common variants to intellectual disability in a sub-isolate of Northern Finland. Nat Communs.

[23] Suokas K (2021) hilmo_identify_episodes (v1.1.0). https://github.com/kmmsks/hilmo_identify_episodes. Accessed 2 Sep 2021

[24] Finnish Environment Institute (2013) Urban-rural classification. https://ckan.ymparisto.fi/dataset/kaupunki-maaseutu-luokitus-ykr. Accessed 4 Apr 2023

[25] Statistics Finland Statistics Finland. In: Origin and background country. https://www.stat.fi/meta/kas/syntypera_ja_ta_en.html. Accessed 11 May 2023

[26] Sundararajan V, Henderson T, Perry C (2004). New ICD-10 version of the Charlson comorbidity index predicted in-hospital mortality. J Clin Epidemiol.

[27] Erlangsen A, Stenager E, Conwell Y (2020). Association between neurological disorders and death by suicide in Denmark. JAMA J AmMed Assoc.

[28] Williams R (2012). Using the margins command to estimate and interpret adjusted predictions and marginal effects. Stand Genomic Sci.

[29] (1940) Mielisairaat ja vajaamieliset. Valtioneuvoston kirjapaino, Helsinki, Finland

[30] The Finnish Institute for Health and Welfare Psychiatric inpatient care, periods of care per 1000 inhabitants. In: Results table - Sotkanet.fi Statistics and Indicator Bank. https://sotkanet.fi/sotkanet/en/taulukko/?indicator=s87yAgA=&region=szZ3tc7UMwQA&year=sy6rsM7R0zUEAA==&gender=t&abs=f&color=f&buildVersion=3.1.1&buildTimestamp=202211091024. Accessed 10 May 2023

[31] Karolaakso T, Autio R, Näppilä T (2021). Contextual and mental health service factors in mental disorder-based disability pensioning in Finland–a regional comparison. BMC Health Serv Res.

[32] Plana-Ripoll O, Pedersen CB, McGrath JJ (2018). Urbanicity and risk of schizophrenia—new studies and old hypotheses. JAMA Psychiat.

[33] Makkonen T, Inkinen T, Rautiainen S (2022). Mapping spatio-temporal variations of shrinkage in Finland. Fennia Int J Geogr.

[34] Hakulinen C, Elovainio M, Arffman M (2020). Employment status and personal income before and after onset of a severe mental disorder: a case-control study. Psychiatr Serv.

[35] Ridley M, Rao G, Schilbach F, Patel V (2020). Poverty, depression, and anxiety: causal evidence and mechanisms. Science.

[36] Suokas K, Koivisto AM, Hakulinen C (2020). Association of income with the incidence rates of first psychiatric hospital admissions in Finland, 1996–2014. JAMA Psychiat.

[37] Hakulinen C, Komulainen K, Suokas K (2023). Socioeconomic position at the age of 30 and the later risk of a mental disorder: a nationwide population-based register study. J Epidemiol Community Health.

[38] Pickett KE, Wilkinson RG (2015). Income inequality and health: a causal review. Soc Sci Med.

[39] Pedersen CB (2015). Persons with schizophrenia migrate towards urban areas due to the development of their disorder or its prodromata. Schizophr Res.

[40] Sariaslan A, Fazel S, D’Onofrio BM (2016). Schizophrenia and subsequent neighborhood deprivation: revisiting the social drift hypothesis using population, twin and molecular genetic data. Transl Psychiatry.

[41] Vaalavuo M, Sihvola M-W (2021). Are the sick left behind at the peripheries? Health selection in migration to growing urban centres in Finland. Eur J Population.

[42] Lankila T, Laatikainen T, Wikström K (2022). Association of travel time with mental health service use in primary health care according to contact type—a register-based study in Kainuu, Finland. BMC Health Serv Res.

[43] Kotavaara O, Nivala A, Lankila T (2021). Geographical accessibility to primary health care in Finland–Grid-based multimodal assessment. Appl Geogr.

[44] Sund R (2012). Quality of the finnish hospital discharge register: a systematic review. Scand J Public Health.

[45] Isohanni M, Mäkikyrö T, Moring J (1997). A comparison of clinical and research DSM-III-R diagnoses of schizophrenia in a Finnish national birth cohort. Soc Psychiatry Psychiatr Epidemiol.

[46] Eurostat (2020) Number of psychiatrists: how do countries compare? https://ec.europa.eu/eurostat/web/products-eurostat-news/-/ddn-20200506-1. Accessed 10 May 2023

[47] Kerminen S, Havulinna AS, Hellenthal G (2017). Fine-scale genetic structure in Finland. G3 Genes Genomes Genet.

[48] Kurki MI, Karjalainen J, Palta P (2023). FinnGen provides genetic insights from a well-phenotyped isolated population. Nature.

[49] Andreassen OA, Hindley GFL, Frei O, Smeland OB (2023). New insights from the last decade of research in psychiatric genetics: discoveries, challenges and clinical implications. World Psychiatry.

[50] Cattarinussi G, Delvecchio G, Sambataro F, Brambilla P (2022). The effect of polygenic risk scores for major depressive disorder, bipolar disorder and schizophrenia on morphological brain measures: a systematic review of the evidence. J Affect Disord.

[51] Machlitt-Northen S, Keers R, Munroe PB (2022). Polygenic risk scores for schizophrenia and major depression are associated with socio-economic indicators of adversity in two British community samples. Transl Psychiatry.

[52] Smeland OB, Bahrami S, Frei O (2020). Genome-wide analysis reveals extensive genetic overlap between schizophrenia, bipolar disorder, and intelligence. Mol Psychiatry.

[53] McCutcheon RA, Keefe RSE, McGuire PK (2023). Cognitive impairment in schizophrenia: aetiology, pathophysiology, and treatment. Mol Psychiatry.

[54] Markota M, Coombes BJ, Larrabee BR (2018). Association of schizophrenia polygenic risk score with manic and depressive psychosis in bipolar disorder. Transl Psychiatry.

[55] Suokas K, Hakulinen C, Sund R (2022). Mortality in persons with recent primary or secondary care contacts for mental disorders in Finland. World Psychiatry.

