# Supplementary material for: Geographical variation in treated psychotic and other mental disorders in Finland by region and urbanicity
Source: Soc Psychiatry Psychiatr Epidemiol. 2023 Jun 13;59(1):37–49. doi: 10.1007/s00127-023-02516-x (PMC10799825; doi:10.1007/s00127-023-02516-x)
Supplement: Supplementary file 1 — Supplementary file1 (PDF 3626 KB) [file 127_2023_2516_MOESM1_ESM.pdf]

Supplementary material

Supplementary Methods 2
Supplementary Figure S1: Regional compositions of Finland 3
Supplementary Table: Prevalence of all mental disorders stratified by the cofactors 4
Supplementary Figure S2: Prevalence ratios of selected mental disorders by place of residence with different levels of adjustment 6
Supplementary Figure S3: Prevalence ratios of selected mental disorders by place of residence in different administrative regions of Finland, compared to the national mean 7
Supplementary Figure S4: Average marginal effects of aggregated region of residence and urbanicity on the prevalence of mental disorders 8
Supplementary Figure S5: Prevalence ratios of selected mental disorders by place of residence in all treatment facilities and inpatient care only 9
Supplementary Figure S6: Prevalence ratios of selected mental disorders by place of residence and place of birth 10
Supplementary Figure S7: Prevalence ratios and incidence rate ratios of inpatient treated mental disorders by place of residence 11
Supplementary Figure S8: Prevalence ratios of selected mental disorders by place of residence, men and women analyzed separately 12
References 13

Supplementary Methods

Assessment of mental disorders from the national registers

The Finnish Hospital Discharge Register (FHDR) has continuous nationwide data since 1969. Finnish Care Register for Health Care (FCRHC) replaced the FHDR in 1994. In the beginning of FHDR, there were separate sub-registers for different hospitals (general/tuberculosis/psychiatric/other), and later, these registers were combined. (Sund, 2012) For the years 1969-1974, the classification of medical specialties is unclear in some of the sub-registries. Hence, people with a history of mental health-related contact with psychiatric inpatient care was evaluated since 1975.

According to the Quality description of the FCRHC, data on specialised outpatient care in the public sector have been collected since 1998, but the data are comparable across time and service providers only from 2006 onwards.(The Finnish Institute for Health and Welfare, 2020) As regional comparison was in the focus of the current study, data since 2006 was used.

Conversion of the ICPC-2 International Classification of Primary Care diagnoses

According to the ICPC-2 manual, "ICPC is based on a simple bi-axial structure: 17 chapters based on body systems on one axis, each with an alpha code, and seven identical components with rubrics bearing a two-digit numeric code as the second axis". (WONCA International Classification Committee (WICC), 2005) Conditions listed in ICPC-2, chapter P: "Psychological" were included in this study. In the Finnish version of ICPC-2, the chapter P is translated to the Finnish equivalent of "mental health" not "psychological" The Finnish version was used throughout the conversion process. (Kvist et al., 2010)

The conversion codes from ICPC-2 to ICD-10 are provided in the ICPC-2 manual and were adopted in this study. ICPC-2 concepts not represented exactly in the ICD-10 were included as a separate group of their own and are not included in the ICD-10 sub-chapter categories.

Supplementary Figure S1: Regional compositions of Finland

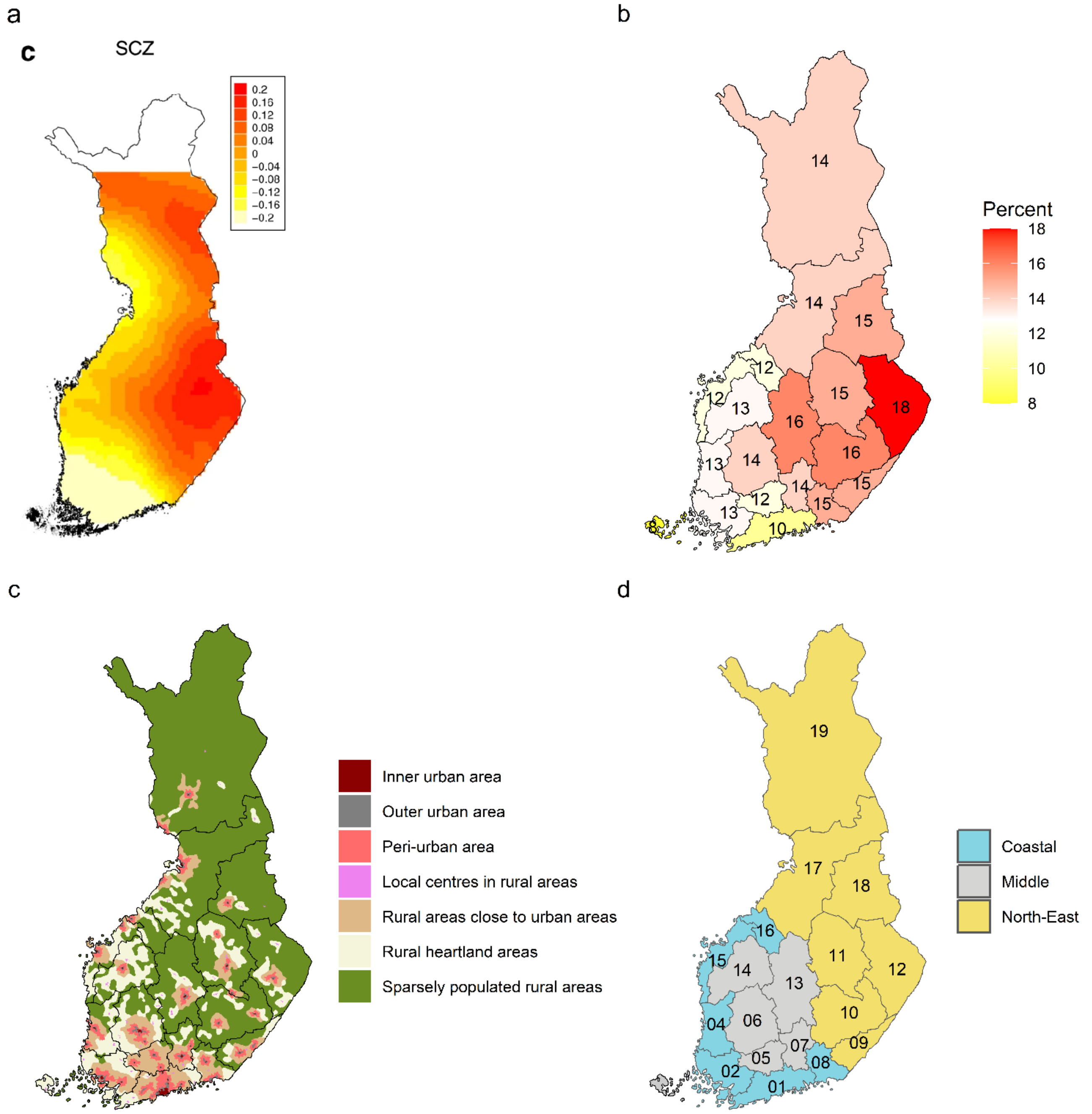

a Regional distribution of schizophrenia polygenic risk-score. Source: Figure 6c in Kurki et al. (2019). Available under a Creative Commons Attribution 4.0 License.

b Low-income earners (percentage of persons at risk of poverty) by region. Source: Statistics Finland (2017)

c Urban-rural classification and administrative regions in Finland. Source: Finnish Environment Institute (2013)

d Administrative regions in Finland and aggregate regions based on the polygenic risk-score distribution in this study. Regions: 01 Uusimaa, 02 Varsinais-Suomi, 04 Satakunta, 05 Kanta-Häme, 06 Pirkanmaa, 07 Päijät-Häme, 08 Kymenlaakso, 09 South Karelia, 10 Etelä-Savo, 11 Pohjois-Savo, 12 North Karelia, 13 Central Finland, 14 South Ostrobothnia, 15 Ostrobothnia, 16 Central Ostrobothnia, 17 North Ostrobothnia, 18 Kainuu, 19 Lapland

Supplementary Table: Prevalence of all mental disorders stratified by the cofactors

	**Whole country**		
	**N (%)**	**Population (%)**	**Prevalence, %**
Gender			
Men	525 338 (43.9)	2 719 032 (49.3)	19.3
Women	672 352 (56.1)	2 793 713 (50.7)	24.1
Person's origin			
Finnish background, born in Finland	1 120 105 (93.5)	5 077 539 (92.1)	22.1
Finnish background, born abroad	14 604 (1.2)	51 385 (0.9)	28.4
Foreign background, born in Finland	11 377 (0.9)	62 572 (1.1)	18.2
Foreign background, born abroad	51 604 (4.3)	321 249 (5.8)	16.1
Living in the region of birth			
No	438 742 (36.6)	1 948 735 (35.3)	22.5
Yes	758 948 (63.4)	3 564 010 (64.7)	21.3
Urbanicity			
Inner urban area	428 063 (35.7)	1 792 079 (32.5)	23.9
Outer urban area	308 122 (25.7)	1 454 480 (26.4)	21.2
Peri-urban area	109 262 (9.1)	608 651 (11.0)	18.0
Local centres in rural areas	73 324 (6.1)	318 981 (5.8)	23.0
Rural areas close to urban areas	71 120 (5.9)	388 748 (7.1)	18.3
Rural heartland areas	120 886 (10.1)	593 821 (10.8)	20.4
Sparsely populated rural areas	57 795 (4.8)	283 320 (5.1)	20.4
Missing	29 118 (2.4)	72 665 (1.3)	40.1
Household income decile			
Non-dwelling	54 208 (4.5)	191 679 (3.5)	28.3
1 (lowest)	202 106 (16.9)	508 435 (9.2)	39.8
2	161 197 (13.5)	525 510 (9.5)	30.7
3	130 224 (10.9)	526 325 (9.5)	24.7
4	113 879 (9.5)	526 957 (9.6)	21.6
5	105 669 (8.8)	527 813 (9.6)	20.0
6	99 042 (8.3)	528 182 (9.6)	18.8
7	91 648 (7.7)	528 805 (9.6)	17.3
8	84 885 (7.1)	529 895 (9.6)	16.0

	Whole country		
	**N (%)**	**Population (%)**	**Prevalence, %**
9	75 044 (6.3)	531 314 (9.6)	14.1
10 (highest)	58 336 (4.9)	532 233 (9.7)	11.0
Data missing	21 452 (1.8)	55 597 (1.0)	38.6
Economic activity			
Employed	411 940 (34.4)	2 327 641 (42.2)	17.7
Unemployed	113 970 (9.5)	296 457 (5.4)	38.4
Aged 0 to 14	133 651 (11.2)	890 376 (16.2)	15.0
Student, pupil	115 233 (9.6)	400 761 (7.3)	28.8
Pensioners and others outside the labour force	422 896 (35.3)	1 597 510 (29.0)	26.5
Charlson comorbidity index			
None	1 003 981 (83.8)	4 880 244 (88.5)	20.6
1-3	182 543 (15.2)	596 759 (10.8)	30.6
≥4	11 166 (0.9)	35 742 (0.6)	31.2

Supplementary Figure S2: Prevalence ratios of selected mental disorders by place of residence with different levels of adjustment

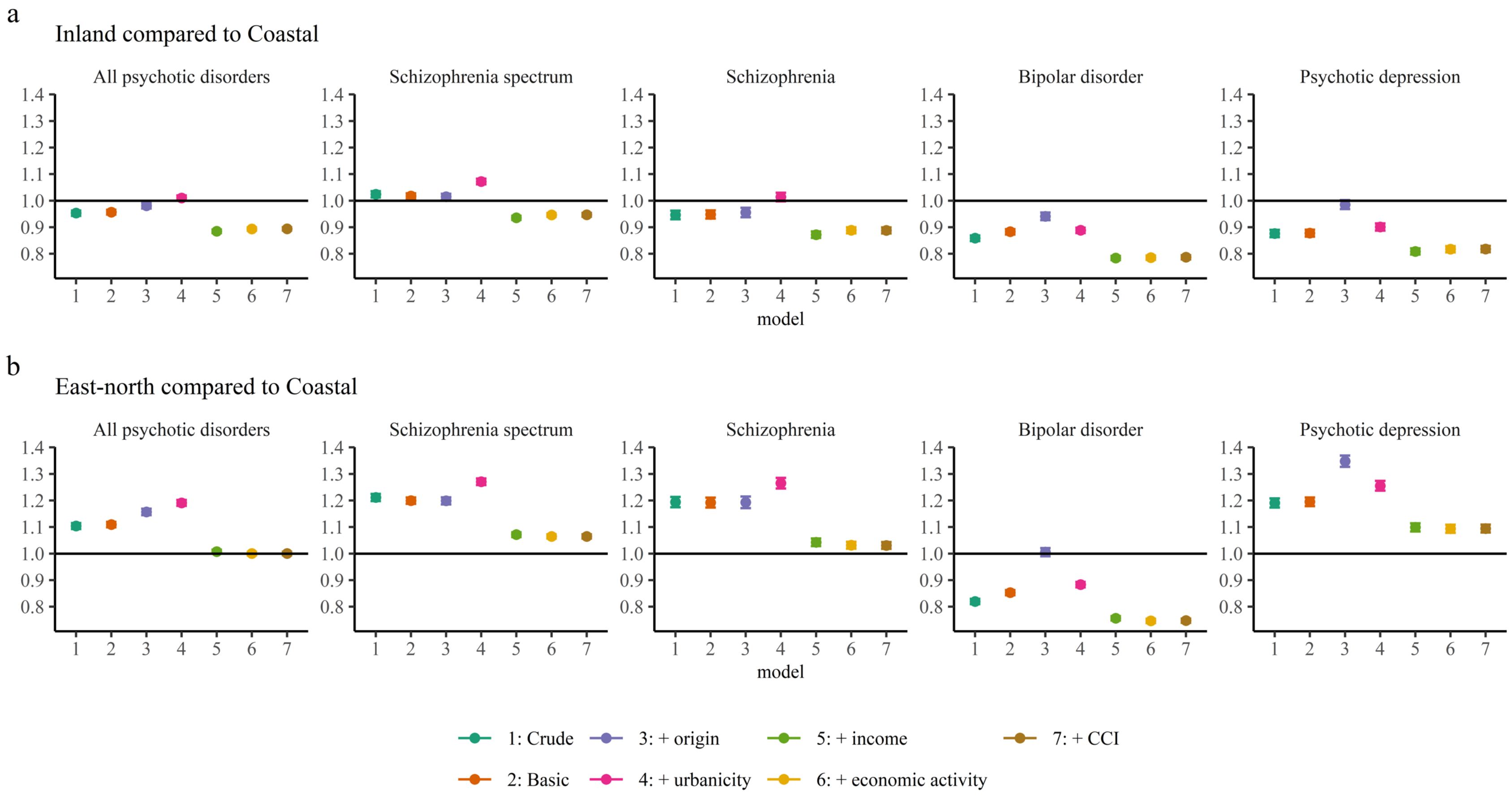

Coastal and eastern and northern regions are described in Figure S1d. In the basic adjustment, prevalence ratios are adjusted for age, gender, and calendar time. Origin refers to persons living in their region of birth. CCI refers to Charlson comorbidity index. Error bars indicate 95% Cis.

Supplementary Figure S3: Prevalence ratios of selected mental disorders by place of residence in different administrative regions of Finland, compared to the national mean

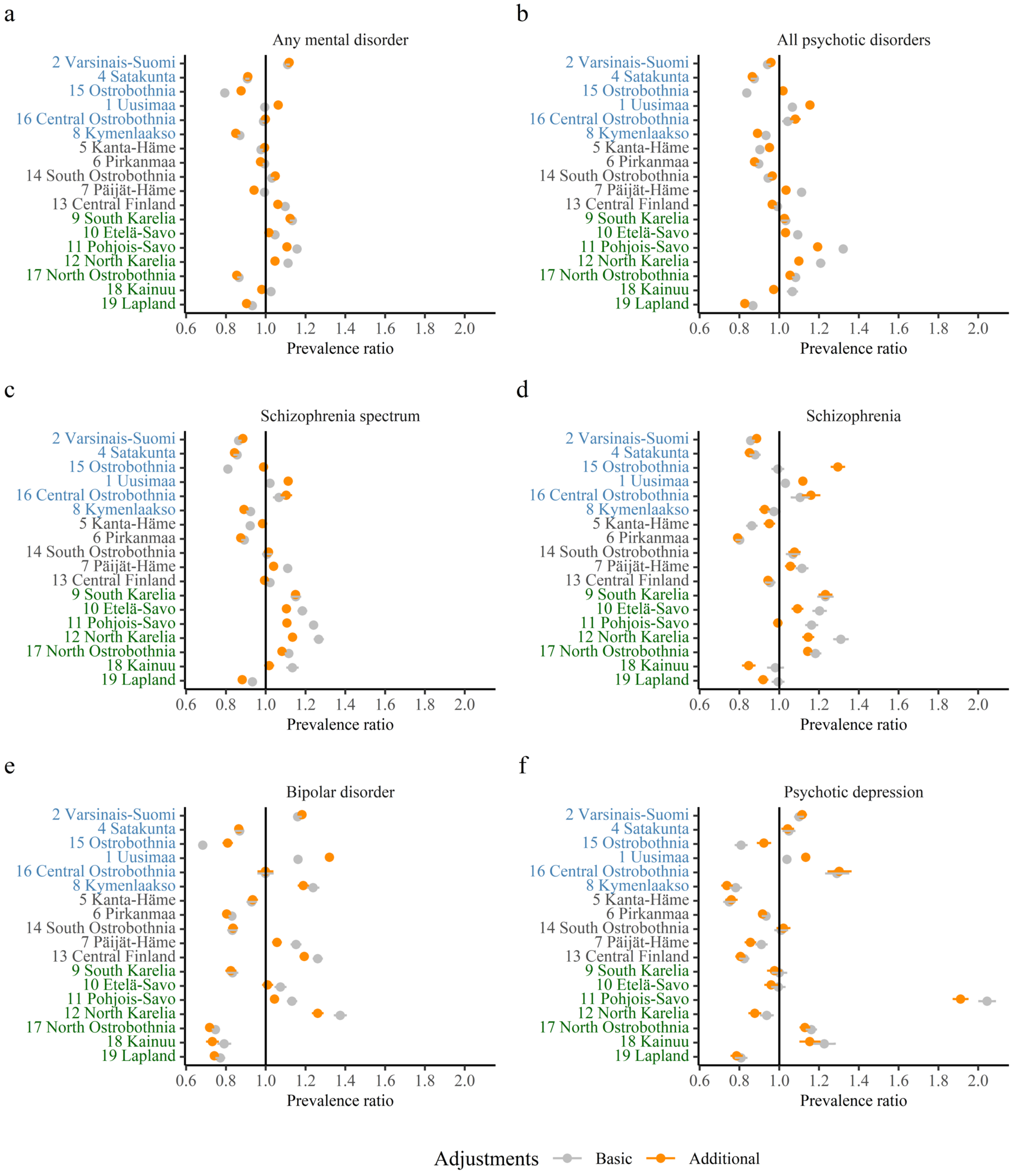

Numbers of the regions correspond to the numbers in the map in Figure S1d. Coastal regions are highlighted in blue, eastern and northern parts in green. In the basic adjustment, prevalence ratios are adjusted for age, gender, and calendar time. In the additional adjustment, prevalence ratios are adjusted for age, gender, calendar time, urbanicity, origin, residence history, household income, economic activity, and Charlson comorbidity index. Error bars indicate 95% CIs.

Supplementary Figure S4: Average marginal effects of aggregated region of residence and urbanicity on the prevalence of mental disorders

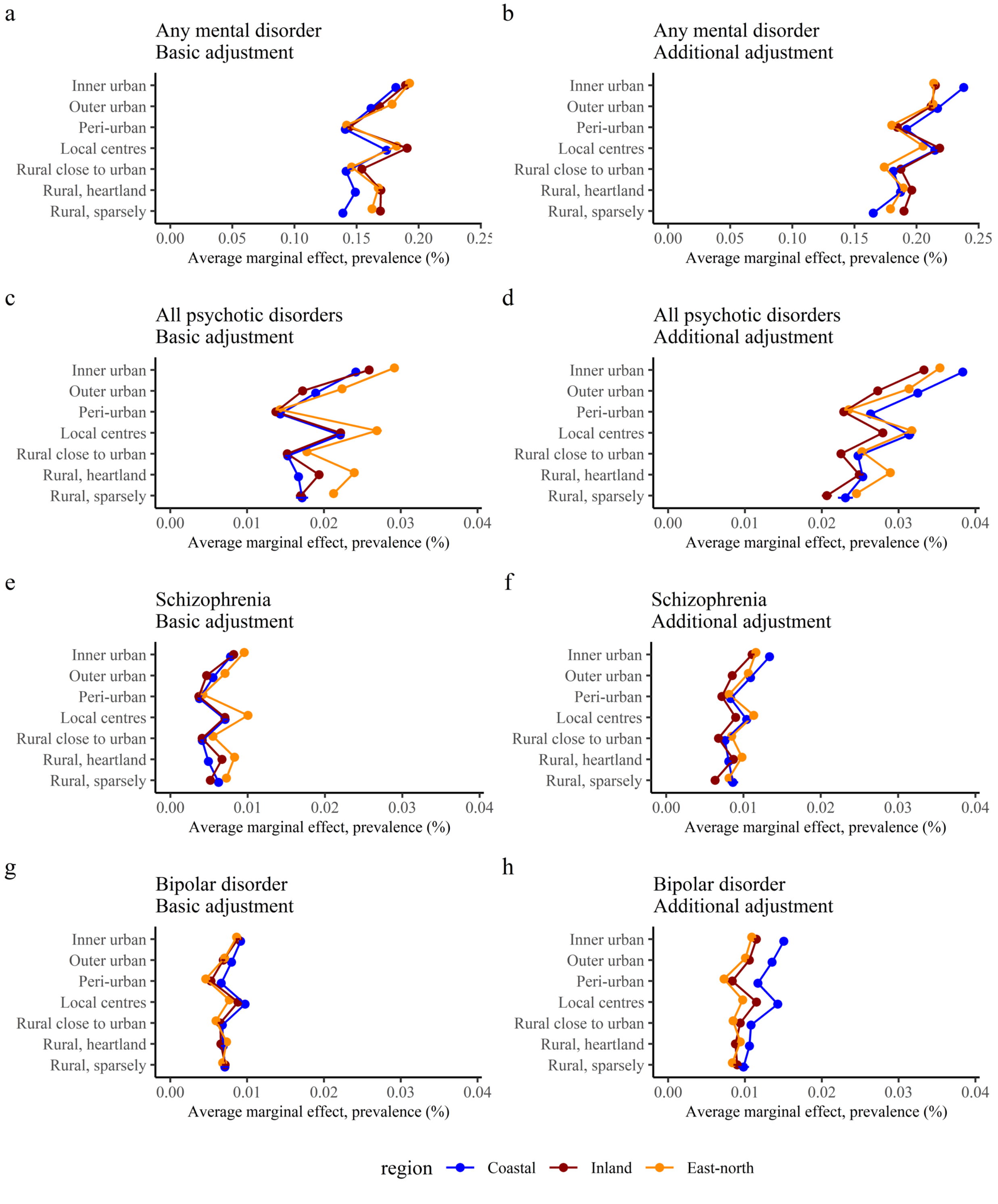

Coastal, Inland, and East-north regions are described in Figure S1d. In the basic adjustment, prevalence ratios are adjusted for age, gender, and calendar time. In the additional adjustment, prevalence ratios are adjusted for age, gender, calendar time, region, origin, residence history, household income, economic activity, and Charlson comorbidity index. Error bars indicate 95% CIs.

Supplementary Figure S5: Prevalence ratios of selected mental disorders by place of residence in all treatment facilities and inpatient care only

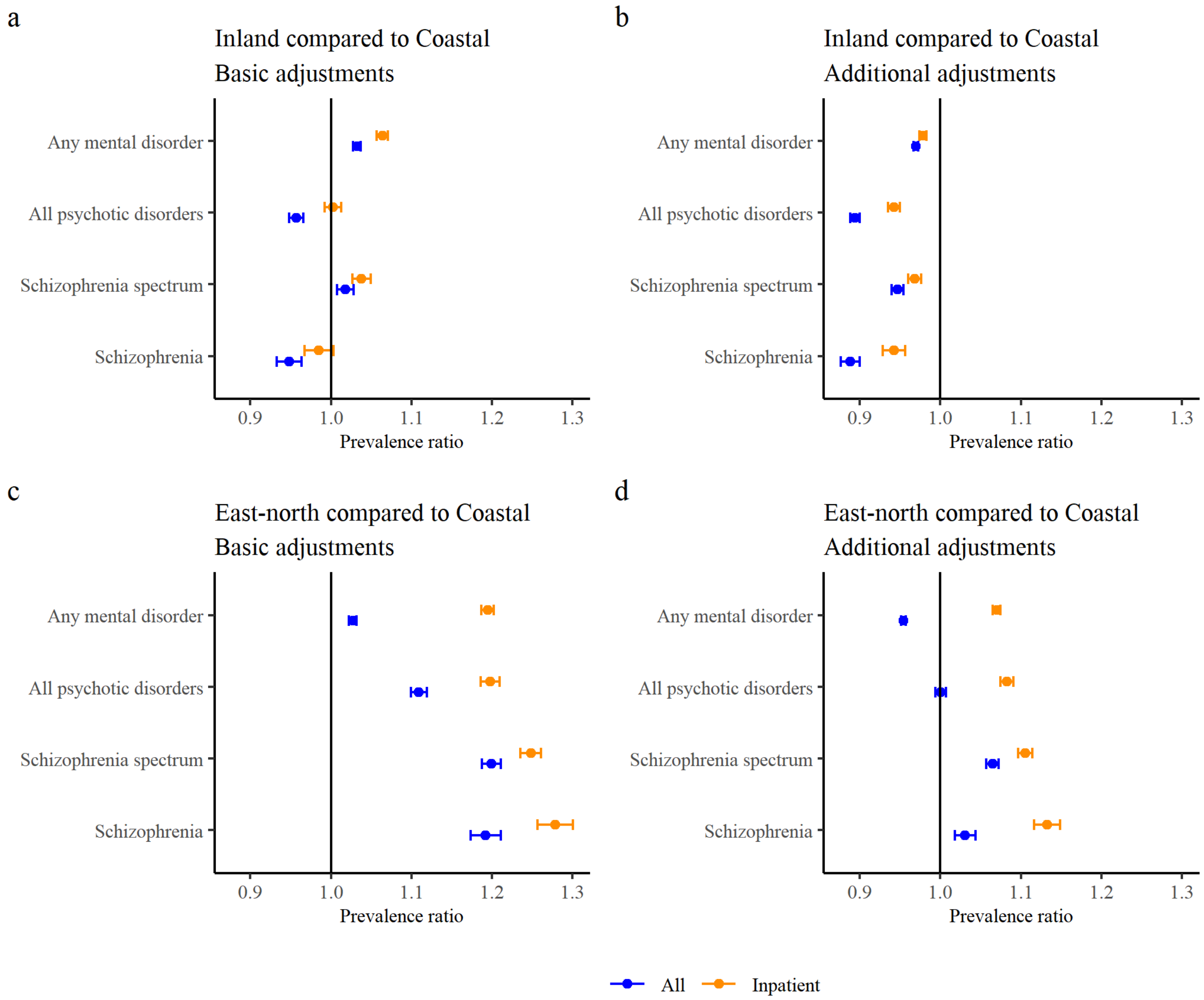

Coastal, Inland, and East-north regions are described in Figure S1d. In the basic adjustment, prevalence ratios are adjusted for age, gender, and calendar time. In the additional adjustment, prevalence ratios are adjusted for age, gender, calendar time, region, origin, residence history, household income, economic activity, and Charlson comorbidity index. Error bars indicate 95% CIs.

Supplementary Figure S6: Prevalence ratios of selected mental disorders by place of residence and place of birth

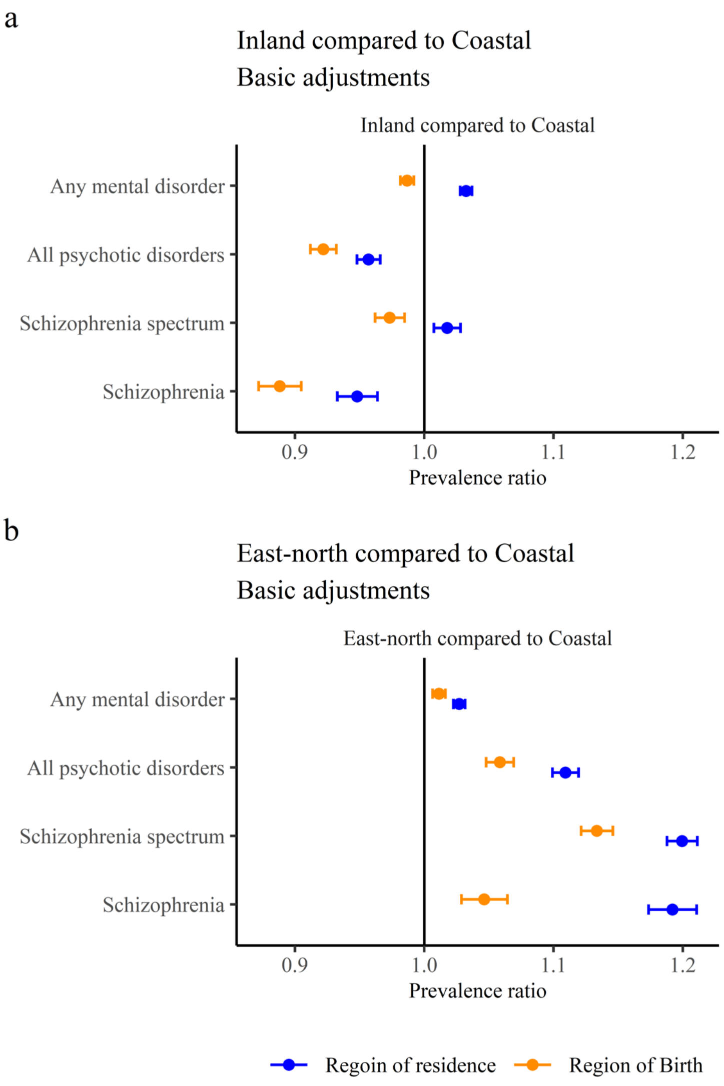

Coastal, Inland, and East-north regions are described in Figure S1d. In the basic adjustment, prevalence ratios are adjusted for age, gender, and calendar time. Error bars indicate 95% CIs.

Supplementary Figure S7: Prevalence ratios and incidence rate ratios of inpatient treated mental disorders by place of residence

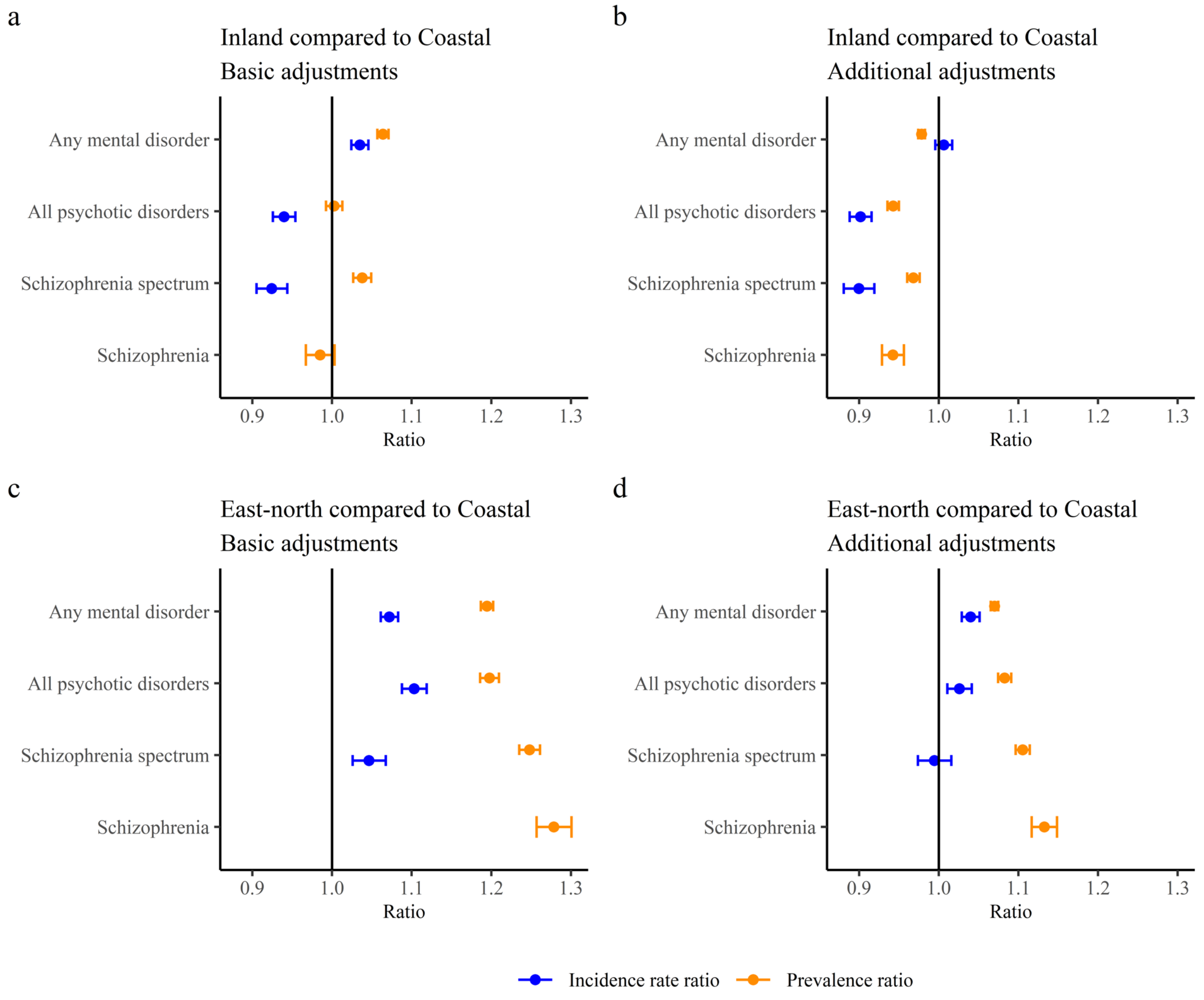

Coastal, Inland, and East-north regions are described in Figure S1d.In the basic adjustment, prevalence ratios are adjusted for age, gender, and calendar time. In the additional adjustment, prevalence ratios are adjusted for age, gender, calendar time, region, origin, residence history, household income, economic activity, and Charlson comorbidity index. Error bars indicate 95% CIs.

Supplementary Figure S8: Prevalence ratios of selected mental disorders by place of residence, men and women analyzed separately

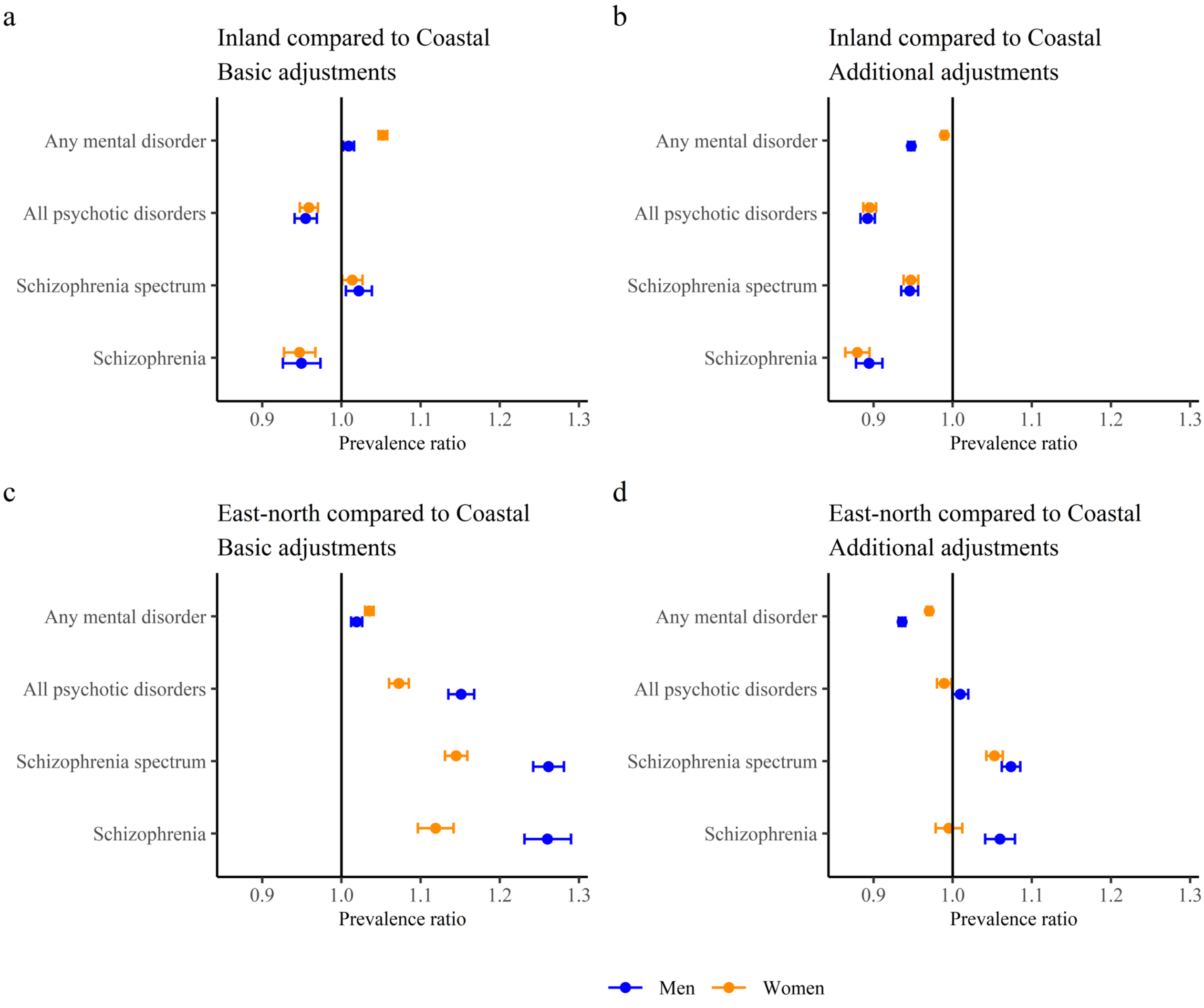

Coastal, Inland, and East-north regions are described in Figure S1d.In the basic adjustment, prevalence ratios are adjusted for age, gender, and calendar time. In the additional adjustment, prevalence ratios are adjusted for age, gender, calendar time, region, origin, residence history, household income, economic activity, and Charlson comorbidity index. Error bars indicate 95% CIs.

References

Finnish Environment Institute. (2013, September 6). *Urban-rural classification*. https://ckan.ymparisto.fi/dataset/kaupunki-maaseutu-luokitus-ykr

Kurki, M. I., Saarentaus, E., Pietiläinen, O., Gormley, P., Lal, D., Kerminen, S., Torniainen-Holm, M., Hämäläinen, E., Rahikkala, E., Keski-Filppula, R., Rauhala, M., Korpi-Heikkilä, S., Komulainen–Ebrahim, J., Helander, H., Vieira, P., Männikkö, M., Peltonen, M., Havulinna, A. S., Salomaa, V., … Palotie, A. (2019). Contribution of rare and common variants to intellectual disability in a sub-isolate of Northern Finland. *Nature Communications 2019 10:1*, *10*(1), 1–15. https://doi.org/10.1038/s41467-018-08262-y

Kvist, M., Savolainen, T., & Suomen Kuntaliitto. (2010). *ICPC-2: Perusterveydenhuollon kansainvälinen luokitus*. Suomen Kuntaliitto.

Statistics Finland. (2017). *Dwelling population's risk of poverty and persistent risk of poverty by Region, Information and Year*. https://pxdata.stat.fi/PxWeb/pxweb/en/StatFin/StatFin__tjt/statfin_tjt_pxt_127z.px/table/tableViewLayout1/

Sund, R. (2012). Quality of the Finnish Hospital Discharge Register: A systematic review. *Scand J Public Health*, *40*(6), 505–515. https://doi.org/10.1177/1403494812456637

The Finnish Institute for Health and Welfare. (2020, October 30). *Erikoissairaanhoito—THL*. https://thl.fi/fi/tilastot-ja-data/aineistot-ja-palvelut/tilastojen-laatu-ja-periaatteet/laatuselosteet/esh

WONCA International Classification Committee (WICC). (2005). *ICPC-2-R: International Classification of Primary Care*. Oxford University Press Inc.
